# Effects of silver diamine fluoride on demineralization protection after a secondary acid challenge

**DOI:** 10.1590/1678-7757-2023-0244

**Published:** 2023-11-03

**Authors:** Mauro A TUDARES, George J ECKERT, Frank LIPPERT

**Affiliations:** 1 Indiana University School of Dentistry Department of Biomedical Sciences and Comprehensive Care Indianapolis Indiana USA Indiana University, School of Dentistry, Department of Biomedical Sciences and Comprehensive Care, Indianapolis, Indiana, USA.; 2 Indiana University School of Medicine Department of Biostatistics and Health Data Science Indianapolis Indiana USA Indiana University, School of Medicine, Department of Biostatistics and Health Data Science, Indianapolis, Indiana, USA.; 3 Indiana University School of Dentistry Department of Cariology, Operative Dentistry and Dental Public Health Indianapolis Indiana USA Indiana University School of Dentistry, Department of Cariology, Operative Dentistry and Dental Public Health, Indianapolis, Indiana, USA.

**Keywords:** Silver diamine fluoride, Remineralization, Enamel, Caries, Transverse microradiography

## Abstract

**Objective:**

This investigation describes the effects of 5% sodium fluoride varnish and 38% silver diamine fluoride on demineralization protection of human enamel lesions of three different severities after a secondary acid challenge.

**Study design:**

Specimens underwent color and enamel surface microhardness change measurements after demineralization and treatment events. Transverse microradiography was conducted following the secondary demineralization.

**Results:**

After treatments, enamel surface microhardness change showed that 24-hour lesions treated with fluoride varnish had less rehardening than 24-hour lesions treated with silver diamine fluoride (p<0.05), whereas 144-hour lesions from both treatment groups showed a beneficial decrease in surface microhardness change that was markedly better in samples treated with silver diamine fluoride (p<0.05). After the secondary demineralization, 24- and 144-hour lesions treated with silver diamine fluoride showed a sustained beneficial decrease in enamel surface microhardness change when compared to fluoride varnish-treated samples of the corresponding lesion severity (p<0.05). Transverse microradiography showed no difference between fluoride varnish- and silver diamine fluoride-treated samples of any corresponding lesion severity, indicating that remineralization in both fluoride varnish- and silver diamine fluoride-treated samples was proportional to each other after a secondary acid challenge.

**Conclusions:**

Using silver diamine fluoride may have comparable benefits to fluoride varnish in mineral loss prevention.

## Introduction

Silver diamine fluoride (SDF) is an alkaline mixture of silver and fluoride ions that stops the action of cariogenic oral bacterial (silver) and hardens the enamel tissue, protecting it from the action of matrix metalloproteinases.^1^ In the caries microenvironment, the SDF-derived fluoride interacts with the mineral components of the enamel and arrests caries.^1^ Additionally, fluoride supports remineralization by diminishing calcium separation from the enamel. Because of its beneficial effects in preventing and arresting dental caries, current research efforts aim at better understanding the action mechanism and applicability of SDF during cariogenic disease progression.^2^

SDF is topically applied in dental clinics to alleviate tooth hypersensitivity and to treat caries lesions that would otherwise require multiple patient-dentist appointments.^3^ SDF application is easy, painless, and more affordable than other options, making it a viable choice to treat caries.^4^ However, an important side effect of SDF refers to dark discoloration or stains on the treated area.

A recent study found that 5% sodium fluoride varnish (FV) might be more appropriate than SDF to treat the less deep enamel lesions that characterize early caries lesions.^5^ Another study that used fluoride and silver nanoparticles separately to control for the effect of particle concentration found that mineral and lesion depth prevention depends on particle concentration and lesion severity.^6^ Regarding SDF, it remains experimentally unknown if the depth of the enamel lesion affects the penetration and action of SDF or if SDF offers additional demineralization protection or remineralization benefits after a second demineralization event. Thus, the overall goal of this study was to evaluate the potential effect of SDF on human enamel discoloration and demineralization protection as a function of baseline lesion severity and assess whether SDF prevents further demineralization, improves remineralization, or both, after a second demineralization event. The impact of SDF on enamel outcomes after a secondary demineralization has not been studied before. Furthermore, no study has evaluated the effects of SDF on enamel caries lesions of varying severities. We hypothesized that SDF treatment will promote enamel remineralization to the same extent as FV, the gold standard for non-surgical caries treatment. Alternatively, we hypothesized that SDF treatment will significantly benefit remineralization beyond that of FV in enamel lesions of greater severity after a second demineralization challenge. To test our hypotheses, we exposed human enamel samples to acid challenges of different durations (1-h/24-h/144-h) to create lesions of varying severities. We subsequently applied SDF topically to compare its effects to FV and deionized water (DIW) as a negative control. After a second demineralization challenge, this study reassessed enamel parameters to determine whether the SDF treatment resulted in greater demineralization protection than that of FV. The underlying guiding question this research aimed to answer refers to whether SDF differs in its comparable ability to FV to prevent further demineralization of enamel caries lesions of varying severities.

## Methodology

### Ethical approval

Local Institutional Review Board approval was obtained for the use of unidentified extracted human teeth (NS0911-07). All procedures were performed in accordance with the ethical standards of the institutional and/or national research committee and with the 1964 Helsinki declaration and its later amendments or comparable ethical standards.

### Study design

**Figure 1:**
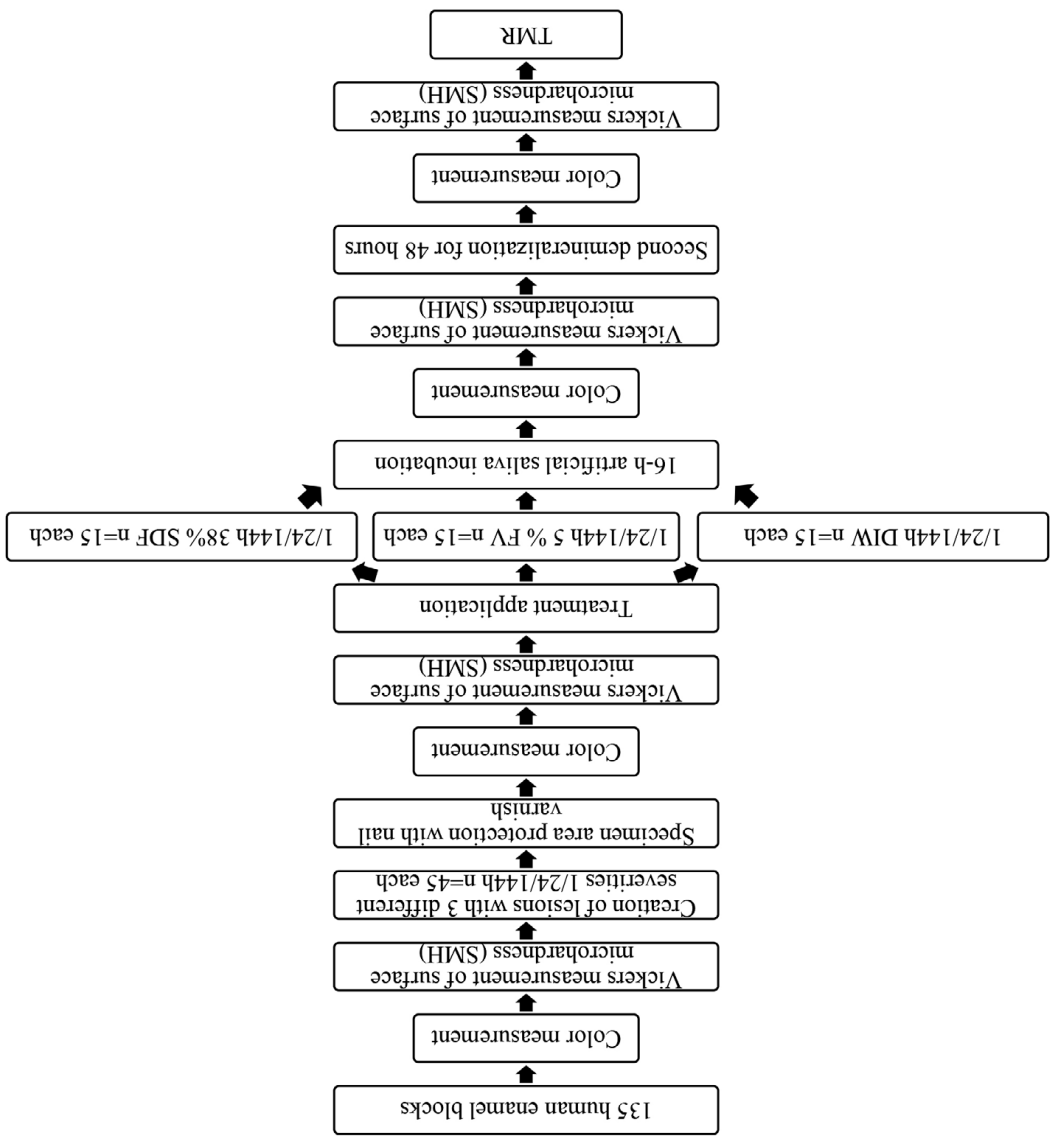
Experimental procedure plan

The experimental sequence of treatments and assessments carried out on the samples is shown in Figure 1 . Initially, all samples were subjected to baseline color and Vickers surface microhardness (SMH) measurements before being grouped in cohorts and exposed to a cariogenic acid solution to create lesions at three different severities according to demineralization time (1-h/24-h/144-h). Subsequently, half of the surface area of each sample was protected with commercially available nail varnish (Sally Hansen Advanced Hard as Nails Nail Polish, USA). Successive color and SMH measurements were performed immediately after the specimen area was protected, after 16-hour incubation using artificial saliva following treatment exposure, and after the second demineralization event. Lastly, mineral loss and lesion depth of the protected side vs. the opposite side were measured using transverse microradiography (TMR). The protected side of the specimen represented the control and the opposite side, the effect of each treatment after the second demineralization as a function of lesion depth and received treatment.

### Specimen preparation

Adult teeth that met the following criteria were used: absence of cracks, fracture lines, white spots, wear, or damage. From this population, 135 teeth were used to obtain the human enamel blocks. Using a low-speed saw (Buehler, Lake Bluff, IL, USA), the crown portion of each tooth was cut into 4×4 mm samples. These constituted our enamel experimental model. Samples needed to have a cubical shape before being used for experimental purposes. To attain this, they were serially ground using a 1200-, 2400-, and 4000-grit silicon carbide grinding paper (Buehler, Lake Bluff, IL, USA) and polished using a Rotopol-31/Rotoforce-4 polishing apparatus (Struers Inc., Cleveland, OH, USA). At all times between each preparation step, samples were stored in a solution of 0.1% w/v thymol in DIW at 4 °C and 100 % relative humidity. Once procured, each sample was attached onto a 1×1-inch acrylic plastic cube using melted wax and a number was assigned to each sample. After the color and SMH baseline measurements, samples were randomly assigned to three groups before the lesions were created (n=45 samples/group). Samples from each lesion group were randomly assigned again into three treatment subcategories (n=15 samples/group) according to Figure 1 .

### Color determination

The color of the enamel samples was measured before any intervention was performed (baseline) and after each acid challenge and treatment events ( Figure 1 ). Only the black and white differences in the tissue were considered.^8 , 9^A Minolta Chroma Meter, model CR-241 (Konica Minolta, Tokyo, Japan), was used. It emitted D65 light while the sample was placed against a white background. Commision Internationale de l’Eclairage L* values were registered for each sample.^7 - 9^ The equipment was calibrated before sample color was measured following manufacturer instructions. Post-intervention color change was measured by the formula: $\Delta L^{*}=L^{*}{}_{\text{intervention }}-L^{*}{}_{\text{baseline }}$

### Vickers surface microhardness (SMH)

SMH was measured after the color assessments ( Figure 1 ). A microhardness tester model 2100 (Wilson Instruments, Norwood, MA, USA) was used. The microhardness tester is equipped with a Vickers diamond that creates an indentation. In total, four indentations were placed at the center of each specimen. The distance between the indentations was set up at 100 µm and 1.96 Newtons of force was applied. Enamel samples with a baseline SMH from 300 to 400 were included in this study.^5 , 8 , 9^ Percent SMH change (%SMHchange) after the first acid challenge, treatment, and 48-hour demineralization were measured by the following formulas: $\%\text{ SMHchange }{post}_{\text{acid }1}=100*(SMH\text{ baseline }-SMH$_,_ $\%\text{ SMHchange }{}_{\text{past treatment }}=100^{*}\left({SMH}_{\text{post acid 1 }}-{SMH}_{\text{post treatment }}\right)/SMH\text{ post acid 1 }$ , and $\%\text{ SMHchange }\text{ post }48h=100*\left({SMH}_{\text{post acid. 1 }}-{SMH}_{\text{post 48h }}\right)/{SMH}_{\text{post acid. }1}$ , respectively. %SMHchange in our *in vitro* approach is interpreted as a loss if it is read after an acid challenge or as a gain or recovery if it is read after a remineralization event, such as a known favorable treatment or an artificial saliva exposure, as described elsewhere.^6^

### Lesion creation

The goal of the first acid challenge was to emulate artificial caries lesions of three different severities. To attain this goal, enamel samples (n=45/group) were immersed in a partially saturated lactic acid solution consisting of 0.1 M lactic acid, 4.1 mM CaCl_2_ × 2 H_2_O, 8.0 mM KH_2_PO_4_, and 0.2% (w/v) Carbopol C907 at 37°C and adjusted to pH=5.0 using KOH^6 , 8^ for 1, 24, or 144 hours. After the immersion period, samples were thoroughly rinsed with DIW. A 48-hour second demineralization challenge (or secondary demineralization) was performed using the same chemical formulation.

### Treatment application and artificial saliva incubation

Subgroups consisting of 15 samples from each lesion severity category were subjected to DIW treatment, 5% sodium fluoride varnish (FV, CavityShield, 3M, St. Paul, MN, USA), or 38% silver diamine fluoride (SDF, Advantage Arrest, Elevate Oral Care, West Palm Beach, FL, USA). DIW (negative control) and SDF were applied by a 2.5-mm diameter micro applicator (Premium Plus International Limited, Hong Kong, China) and rubbed onto the sample surface for 10 seconds, let to air dry for 1 minute and rinsed briefly with DIW. FV was applied using the manufacturer supplied micro-brush for 10 seconds and rinsed briefly with DIW. Residual FV was manually removed from the sample surface using chloroform. After exposure to individual treatments, samples were incubated at 37 °C for 16 hours in an artificial saliva solution containing 1.5mM CaCl_2_×٢ H_2_O; 0.9 mM KH_2_PO_4; _130 mM KCL; 20 mM HEPES; 3.1 mM NaN_3_. The pH was adjusted to 7.0 using KOH.^10^

### Transverse microradiography (TMR)

All samples were subjected to complete sectioning across their middle area and transversally to the nail varnish area to obtain one 100-µm section using a Silverstone-Taylor Hard Tissue Microtome (Scientific Fabrications Laboratories, USA) as previously described.^9^ Prior to TMR analysis, the protected and treated side of each 100-µm section needed to be clearly defined. This was achieved by the microscopical analysis of each section. Integrated mineral loss and lesion depth were analyzed using a window of 400 × 400 µm representing the area under the nail varnish (protected area) and the treatment area (unprotected area). The 100-µm sections were fixed onto a 2”×2” glass panel using plastic wrap. The aluminum step wedge calibration processor captured images in 14 steps. Then, a Thermo-Kevex PXS5 X-ray source was used to capture the sample images. A TMR-D 5.0.01 software (Inspektor Research Systems, Amsterdam, NL) was used for the initial reading of the files before analysis with TMR2006, v.3.0.0.18.^6 , 9^

### Statistical analysis

Calculations were based on a 5% significance level and two-sided tests, using the t-test after the initial demineralization event and two-way ANOVA with the factors ‘baseline lesion severity’ and ‘treatment.’ Statistical analyses were performed on JMP, version 16.2. SAS Institute Inc. (Cary, NC, USA 1989-2022).

## Results

**Figure 2:**
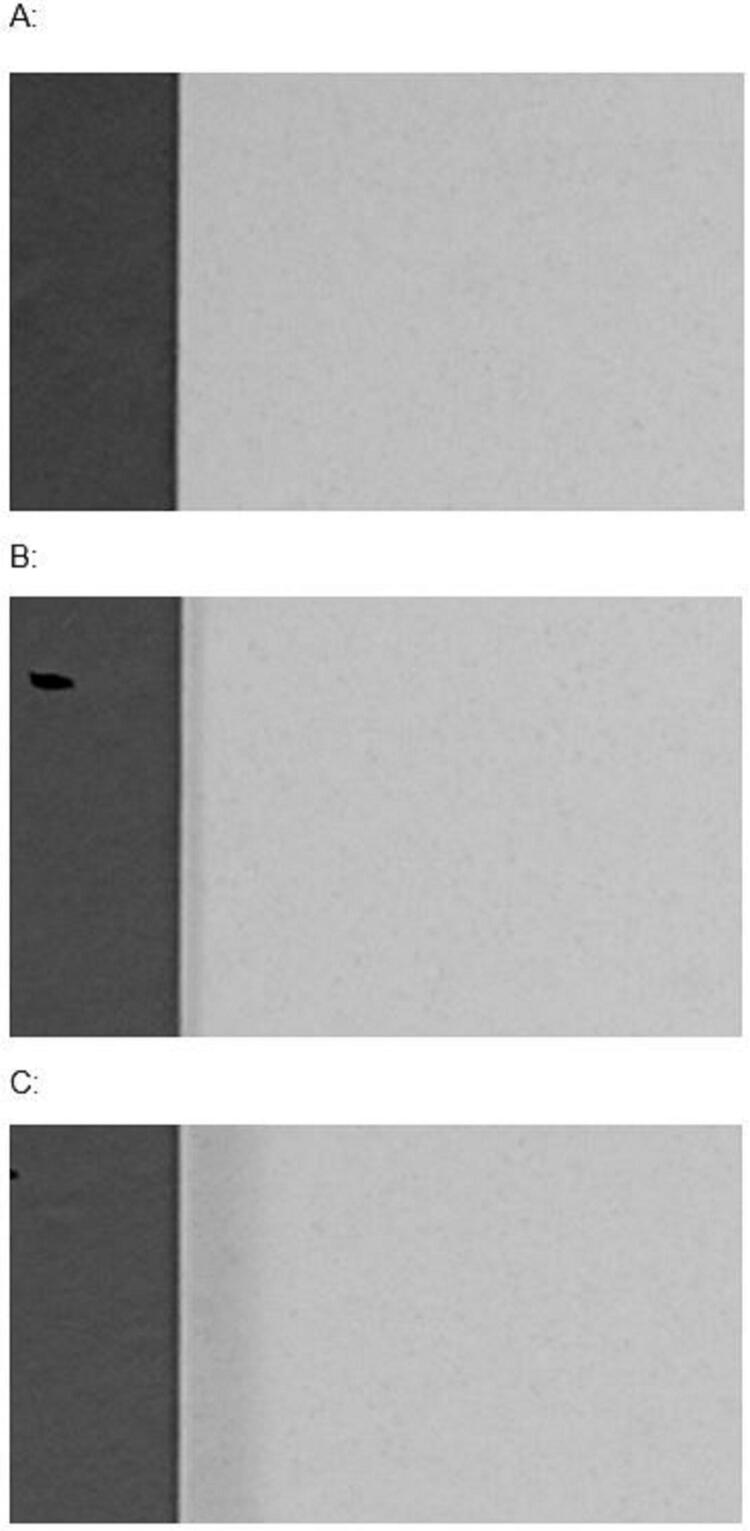
Microradiographic image of the representative baseline caries lesions. A: 1-h lesion, B: 24-h lesion, C:144-h lesion

Figure 2 shows the representative baseline lesions for each severity, in which 1-h demineralization resulted in no radiographically visible demineralization, whereas 24- and 144-h lesions displayed characteristics of typical subsurface caries-like lesions.

### Color assessment

The results of the two-way ANOVA determined a statistically significant interaction ( *p* <0.0001) between initial lesion severity and treatment on color after treatment and the secondary demineralization events. Table 1 shows ΔL* data after the initial lesion creation, post-treatment, and after second demineralization. Acid challenge exposure time positively affected ΔL* values ( *p* <0.0001) and 144-h affected them the most. ΔL* after treatment showed that SDF in the 24- and 144-h lesion groups similarly increased the dark staining of samples ( *p* =0.074), negatively affecting ΔL* values when compared to other treatment groups. After the 48-hour demineralization, SDF-treated groups had ΔL* values significantly lower than the corresponding DIW and FV groups at initial acid exposure times.

**Table 1:** Color data (L*) of sound enamel specimens by lesion severity and treatment plan and color change data (ΔL*) throughout the study. Positive ΔL* values represent increased whiteness of the specimens. Data are shown as means ± standard deviations. Different letters represent statistically significant differences between groups in each experimental step (Sig column)

		Sound enamel		Lesion baseline		Post-treatment application		Post-secondary demineralization	
Treatment	Lesion severity	L*	Sig	ΔL*	Sig	ΔL*	Sig	ΔL*	Sig
DIW	1-h	71.8±2.4	A	1.5±1.3	C	-6.7± 3.6	BC	-2.1±4.0	B
	24-h	71.2±3.5	A	3.7±2.7	B	-3.7±2.7	B	-0.3±2.9	B
	144-h	72.3±2.2	A	12.7±3.2	A	10.7±2.4	A	9.8±2.7	A
FV	1-h	71.0±3.7	A	1.4±2.9	C	-9.7±2.1	CD	-13.8±2.9	CD
	24-h	73.0±2.3	A	3.8±2.8	B	-7.1±4.4	BCD	-9.4±3.7	C
	144-h	72.8±3.5	A	14.7±2.6	A	9.1±3.0	A	5.7±3.0	A
SDF	1-h	72.2±3.3	A	0.5±1.6	C	-11.1±3.8	D	-18.2±3.7	D
	24-h	71.8±2.5	A	4.3±3.1	B	-29.2±4.6	E	-40.4±4.4	E
	144-h	71.9±3.5	A	14.0±3.4	A	-26.8±5.8	E	-43.6±8.2	E

### Surface microhardness

The results of the two-way ANOVA determined a statistically significant interaction between initial lesion severity and treatment on SMH ( *p* <0.0001) after treatment and secondary demineralization. Table 2 shows the %SMHchange_post acid 1_, %SMHchange_post treatment_, and %SMHchange_post 48h_. Acid challenge exposure time caused a time-dependent increase in %SMHchange ( *p* <0.0001). The 24-h lesion comparison between FV and SDF treatment groups evinced an increase in %SMHchange_post treatment _for FV-treated lesions when compared to SDF-treated lesions (7.2 vs. −67.1; *p* <0.05). The 144-h lesion comparison between FV and SDF treatment groups showed a decrease in %SMHchange_post treatment _for both FV- and SDF-treated samples (−3.2 vs. −43.5, *p* <0.05). After the 48-hour redemineralization, the %SMHchange_post 48h _between FV- and SDF-treated groups decreased for both 24- (−6.4 vs. –33.1; *p* <0.05) and 144-h (−4.4 vs. −47.2; *p* <0.05) lesions.

**Table 2:** Surface microhardness data (SMH) of sound enamel specimens by treatment group and lesion severity and % surface microhardness change data (%SMH change) throughout the study. Positive %SMH change values represent greater softening of the tissue, whereas negative %SMH change values indicate rehardening. Data are shown as means ± standard deviations. Different letters represent statistically significant differences between groups in each experimental step (Sig column)

		Sound enamel		Lesion baseline		Post-treatment application		Post-secondary demineralization	
Treatment	Lesion severity	SMH	Sig	%SMH change	Sig	%SMH change	Sig	%SMH change	Sig
DIW	1-h	350±18	A	5±6	C	-4±7	AB	60±9	A
	24-h	364±16	A	66±3	B	-25±11	C	7±8	B
	144-h	350±18	A	84±3	A	-12±20	BC	-7±16	B
FV	1-h	341±26	A	4±11	C	5±6	A	5±8	B
	24-h	354±24	A	65±6	B	7±7	A	-6±14	B
	144-h	354±12	A	85±4	A	-3±14	AB	-4±17	B
SDF	1-h	348±13	A	5±6	C	-6±6	AB	-4±11	B
	24-h	344±16	A	68±4	B	-67±13	E	-33±16	C
	144-h	343±20	A	85±4	A	-43±20	D	-47±35	C

### Transverse microradiography

The two-way ANOVA analysis found no statistically significant interaction between initial lesion severity and treatment on integrated mineral loss (ΔΔZ). However, effect analysis showed that initial lesion severity influenced mineral loss ( *p* <0.0001), whereas the received treatment failed to do so. Accordingly, the effect of initial lesion severity on mineral loss prevention after the 48-h demineralization was significant in 144-h lesions when compared to 1- or 24-h lesions. As mentioned, mineral loss prevention as a result of received treatment showed no statistically significant differences between DIW, FV, and SDF treatments ( *p* >0.05, table 3 ). Of note, 144-h samples treated with either FV or SDF underwent more remineralization (166 and 405 vol%min × µm, respectively), whereas corresponding samples treated with DIW had a ΔΔZ of −87, indicating greater mineral loss in samples treated with the negative control treatment. For lesion depth change ΔL, two-way ANOVA analysis found no statistically significant interaction between initial lesion severity and treatment. However, effect analysis showed that initial lesion severity and treatment had an autonomous influence on lesion depth ( *p* <0.01 and *p* <0.05, respectively, Table 3 ). After 48-h demineralization, samples of all lesion severities treated with DIW, as well as 1- and 24-h samples treated with either FV or SDF, showed positive ΔL values, representing deeper lesions. Moreover, 144-h samples treated with either FV or SDF showed negative ΔL values, representing shallower enamel lesions. Treatment effects on ΔL between SDF and FV were similar.

**Table 3:** Mineral loss Δ(Δ)Z [vol%min×µm] and lesion depth (Δ)L [µm] as measured by transverse microradiography (TMR) after samples underwent primary (lesion baseline) and secondary demineralization challenges. Negative ΔΔZ values represent further mineral loss. Positive (Δ)L values represent further increase in lesion depth. Data are shown as means ± standard deviations. Different capital letters represent statistically significant differences between groups in each experimental step (Sig column). Different lowercase letters represent statistically significant differences based on received treatment

		Lesion baseline				Post-secondary demineralization			
		Mineral loss		Lesion depth		Mineral loss		Lesion depth	
Treatment	Lesion severity	ΔZ	Sig	L	Sig	ΔΔZ	Sig	ΔL	Sig
DIW	1-h	188±211	C	6±5	Ca	-243±305	Ba	15±12	Aa
	24-h	608±435	B	27±24	Ba	-155±455	Ba	8±23	Aa
	144-h	1570±266	A	68±11	Aa	-87±494	Aa	8±18	Ba
FV	1-h	221±182	C	5±2	Ca	-15±174	Ba	0±2	Ab
	24-h	481±153	B	19±4	Ba	-87±242	Ba	5±8	Ab
	144-h	1605±317	A	73±16	Aa	166±520	Aa	-11±19	Bb
SDF	1-h	149±200	C	4±2	Ca	-78±311	Ba	3±7	Aab
	24-h	506±166	B	21±8	Ba	-53±338	Ba	8±12	Aab
	144-h	1674±542	A	72±22	Aa	405±376	Aa	-6±25	Bab

## Discussion

This study used human enamel samples to test the effect of SDF on enamel demineralization protection as a factor of lesion severity and after a second demineralization. We were interested in comparing any SDF effects on enamel lesions to FV, the current gold standard used for non-surgical caries management. Standardized enamel samples underwent artificial caries lesion creation following three time exposures to a cariogenic challenge and the subsequent application of one of the following treatments: DIW as a negative control, 5% FV, or a 38% SDF solution. After submerging the samples for 16 hours in artificial saliva, samples underwent a second acid challenge consisting of a 48-hour immersion in a partially saturated lactic acid solution that resembled that to create the initial caries lesions. For the analysis of ΔL*, %SMHchange, mineral loss (Δ(Δ)Z), and lesion depth ((Δ)L), we determined that the most logical approach was to compare lesions of the same severity between different treatments, in which the severity of the lesion is defined by the immersion time during the first acid challenge. For instance, we ignored the comparison of the effect of a treatment in a 1-h lesion vs. the effect of a different treatment in a 24- or a 144-h lesion as depth affects the porosity of the lesions due to the acid challenge.

The initial acid challenge duration in the partially saturated lactic acid solution established the three severity levels of the enamel lesions (1-, 24-, and 144-h). Exposure to the first acid challenge caused the ΔL* of the samples to change in a time-dependent manner, with 144-h lesion samples having greater ΔL* values than the ١- and ٢٤-h lesions ( Table 1 ), indicating that the enamel became whiter with prolonged immersion in the partially saturated lactic acid solution, as with decalcified white spot lesions in cases with persisting poor oral hygiene.^11^ Likewise, prolonged acid exposure times negatively affected the %SMH change, depicted in our data as a percentage change increase or enamel softening ( *p* <0.0001). Taken together, ΔL* and %SMH change after the first acid challenge exposure experimentally validated the artificial caries creation in this study.

After treatment, this study found no differences in ΔL* between 1-h lesions treated with FV or SDF ( *p* =0.296), potentially indicating the low penetration of SDF into superficial caries lesions. Consistent with our results, other studies on deciduous teeth found that silver ions penetrated demineralized enamel by following the natural direction of rods and rod sheaths and failed to penetrate sound enamel.^12^ After the secondary 48-h demineralization, ΔL* remained similar between 1-h lesions treated with FV or SDF ( *p* >0.05), possibly indicating that 1-h lesions are insufficiently demineralized to enable sufficient silver ion penetration and color darkening. After treatment and the secondary 48-h demineralization, 24-h lesions treated with SDF showed darker ΔL* values than 24-h lesions treated with FV ( *p* <0.05). It can be inferred that a 24-hour exposure to the cariogenic solution in this study is sufficient for silver ions to penetrate the demineralized enamel, thus defining a true demineralized lesion.

Regarding surface microhardness, positive %SMHchange values indicate that the enamel softened or that it underwent more non-beneficial hardness change. After treatment, increased softening of the 1- and 24-h FV-treated samples may suggest that parts of the sticky solution remained on the surface of samples, preventing deeper segments from benefiting from the exposure to FV. Moreover, 24- and 144-h lesions treated with SDF benefited the most by showing hardening consistent with deep penetration of silver ions into the enamel. After the 48-h acid challenge, 24- and 144-h lesions treated with SDF showed improved hardening than the corresponding FV-treated samples.

TMR analysis quantified mineral loss and gain change after the 48-h acid challenge. All DIW-treated lesions experienced mineral loss irrespective of lesion depth. Likewise, both 1- and 24-h samples treated with SDF experienced mineral loss change but to the same extent as corresponding samples treated with FV. Instead, 144-h samples treated with FV or SDF had comparable mineral gain change and shallower lesions ( table 3 ).

Remineralization within FV-treated lesions was non-linear. Instead, 24-h lesions showed more mineral loss change than 1-h lesions. This might be the consequence of acid exposure affecting the mineral content and density differently on the surface of the lesions than on the body.^13^ TMR, albeit destructive, was a valuable method to measure mineralization gain change after the secondary 46-h demineralization in our enamel samples, as it happened in the 144-h samples treated with FV or SDF. However, it is unable to measure real-time demineralization and remineralization as other methods such as µCT.^14^ Using µCT could have been valuable to better understand the dynamics of mineral dissolution and gain in our model, but it lacks the sensitivity of TMR.

One advantage of our experimental method is that we used human enamel, naturally characterized by chemical gradients that are important for enamel function and strength during physiological conditions and disease onset and progression.^15^ Although we used methods to create lesions that have been used before, some technical limitations remain in our experimental approach: the application of the FV in our study followed manufacturer instructions but the product leaves a sticky residue that had to be artificially removed with chloroform. It is unknown to us whether the chloroform influenced the action of the FV on the enamel. A previous study^16^ found that chloroform, a solvent, decreases Vickers microhardness in a time-dependent manner when applied to enamel for 5 or 15 minutes compared to baseline. Although we only used chloroform for a few seconds, we are unable to rule out any unanticipated effect of the solvent on our FV-treated samples.

Another limitation of our study is that the experimental model we used is free of bacterial deposits or accumulation that normally characterize enamel and determines caries formation.^17^ By lacking a biofilm, our model fails to exactly mimic the physiological conditions of demineralized enamel in the oral cavity. An additional limitation is that our experimental approach is unable to infer how SDF progressively affects caries lesion development because it was applied at very precise time points after the creation of the lesions. Future studies should also consider the use of pH cycling models to mimic *in vivo* caries more closely under laboratory settings. However, our model measured the extent FV and SDF reduced lesion depth and lesion remineralization. Lastly, our study ignored how to alleviate the stain stemming from SDF application in 24- and 144-h lesions. Future studies could explore any synergistic effect of different whitening products and toothbrushing on the stains created by SDF as abrasion can make stains less visible.^7^

This laboratory investigation is important because it evaluates the effects of SDF on enamel demineralization protection after a secondary acid challenge. The overall major finding of this study is that SDF provides a remineralization advantage in more demineralized, experimental caries lesions, as measured by both the SMH and TMR, compared to more superficial lesions that also received the SDF treatment. TMR analysis showed that SDF delivers this benefit predominantly on 144-h lesions but not in 1- and 24-h lesions. In other words, although we failed to prove that SDF would have a stronger remineralization benefit that FV (our alternative hypothesis), we found that initial lesion severity is a significant factor affecting tissue response to SDF, with deeper lesions receiving a greater benefit from SDF than more superficial lesions. Therefore, using SDF in the clinic may benefit caries management by protecting against mineral loss, especially in advanced caries lesions, despite its local dark-coloring effects.

## Conclusion

Our results indicate that SDF may benefit the treatment of caries to prevent continuous demineralization in more advanced enamel caries lesions or in cases of recurrent or difficult to deter noxious cariogenic stimulus.

## References

[1] Mei ML, Lo EC, Chu CH (2018). Arresting dentine caries with silver diamine fluoride: what's behind it?. J Dent Res.

[2] Machiulskiene V, Campus G, Carvalho JC, Dige I, Ekstrand KR, Jablonski-Momeni A (2020). Terminology of dental caries and dental caries management: Consensus Report of a Workshop Organized by ORCA and Cariology Research Group of IADR. Caries Res.

[3] Horst JA, Ellenikiotis H, Milgrom PL (2016). UCSF protocol for caries arrest using silver diamine fluoride: rationale, indications and consent. J Calif Dent Assoc.

[4] Bridge G, Martel AS, Lomazzi M (2021). Silver diamine fluoride: transforming community dental caries program. Int Dent J.

[5] Alcorn A, Al Dehailan L, Cook NB, Tang Q, Lippert F (2022). Longitudinal in vitro effects of silver diamine fluoride on early enamel caries lesions. Oper Dent.

[6] Aldhaian BA, Balhaddad AA, Alfaifi AA, Levon JA, Eckert GJ, Hara AT (2021). In vitro demineralization prevention by fluoride and silver nanoparticles when applied to sound enamel and enamel caries-like lesions of varying severities. J Dent.

[7] Alshara S, Lippert F, Eckert GJ, Hara AT (2014). Effectiveness and mode of action of whitening dentifrices on enamel extrinsic stains. Clin Oral Investig.

[8] Sorkhdini P, Gregory RL, Crystal YO, Tang Q, Lippert F (2020). Effectiveness of in vitro primary coronal caries prevention with silver diamine fluoride - chemical vs biofilm models. J Dent.

[9] Sorkhdini P, Crystal YO, Tang Q, Lippert F (2021). The effect of silver diamine fluoride in preventing in vitro primary coronal caries under pH-cycling conditions. Arch Oral Biol.

[10] Lippert F (2017). Effect of enamel caries lesion baseline severity on fluoride dose-response. Int J Dent.

[11] Khoroushi M, Kachuie M (2017). Prevention and treatment of white spot lesions in orthodontic patients. Contemp Clin Dent.

[12] Li Y, Liu Y, Psoter WJ, Nguyen OM, Bromage TG, Walters MA (2019). Assessment of the silver penetration and distribution in carious lesions of deciduous teeth treated with silver diamine fluoride. Caries Res.

[13] Devadiga D, Shetty P, Hegde MN (2022). Characterization of dynamic process of carious and erosive demineralization - an overview. J Conserv Dent.

[14] Davis GR, Mills D, Anderson P (2018). Real-time observations of tooth demineralization in 3 dimensions using X-ray microtomography. J Dent.

[15] DeRocher KA, Smeets PJ, Goodge BH, Zachman MJ, Balachandran PV, Stegbauer L (2020). Chemical gradients in human enamel crystallites. Nature.

[16] Rotstein I, Cohenca N, Teperovich E, Moshonov J, Mor C, Roman I (1999). Effect of chloroform, xylene, and halothane on enamel and dentin microhardness of human teeth. Oral Surg Oral Med Oral Pathol Oral Radiol Endod.

[17] Sanz M, Beighton D, Curtis MA, Cury JA, Dige I, Dommisch H (2017). Role of microbial biofilms in the maintenance of oral health and in the development of dental caries and periodontal diseases. Consensus report of group 1 of the Joint EFP/ORCA workshop on the boundaries between caries and periodontal disease. J Clin Periodontol.

